# Audit of the functional preparedness of the selected military hospital in response to incidents and disasters: participatory action research

**DOI:** 10.1186/s12873-022-00728-z

**Published:** 2022-10-13

**Authors:** Esmail Heidaranlu, Mehdi Amiri, Mohammad Mehdi Salaree, Forogh Sarhangi, Yaser Saeed, Asghar Tavan

**Affiliations:** 1 Trauma Research Center, Nursing Faculty, Baqiyatallah University of Medical Sciences, Tehran, Iran; 2 Trauma Research Center, Nursing Faculty, Baqiyatallah University of Medical Sciences, Tehran, Iran; 3 Health Research Center, life style institute, Nursing faculty, Baqiyatallah University of Medical Sciences, Tehran, Iran; 4 Disasters and Emergencies Research Center, Institute for Futures Studies in Health, Kerman University of Medical Sciences, Kerman, Iran

**Keywords:** Functional preparedness, Hospital, Disasters, Audit, Action research, Emergency, Incidents

## Abstract

**Introduction:**

Since hospitals play an important role in dealing with disaster victims, this study was conducted to audit the functional preparedness of the selected military hospital in response to incidents and disasters.

**Materials and methods:**

This applied action research was conducted in all wards of a military hospital from September 2020 to September 2021. The functional preparedness of the hospital was assessed using a functional preparedness checklist containing 17 domains and the weaknesses of the hospital were identified. Then, during the hospital audit cycle, a plan was developed to improve work processes and the functional preparedness of different wards of the hospital in response to incidents and disasters using the FOCUS-PDCA model. The functional preparedness of the hospital was compared before and after the intervention and analyzed using SPSS22.

**Results:**

The relative mean score of hospital preparedness in response to disasters was 508 out of 900 (56.44%) before the intervention, which was moderate. The relative mean score of the hospital preparedness in response to disasters was 561 (63.63%) after the intervention, which was good. The highest preparedness was related to risk assessment (85%) and the lowest preparedness was related to victims’ dead bodies (44%).

**Conclusion:**

Considering the effect of action research on improving the hospital’s functional preparedness in response to disasters, other healthcare facilities are encouraged to incorporate auditing into their work plans.

## Introduction

In disasters, a large number of injured resorts to hospitals for treatment, so hospitals and health centers are among the first to provide optimal and timely healthcare services, which can reduce mortality rates and rescue the injured. Therefore, a hospital is considered a valuable center for disaster management activities, and its preparedness to deal with the devastating consequences of disaster is not hidden from anyone [1]. As hospitals encounter overlapping jobs and inconsistencies in operations during disasters, preparedness is a top concern for the majority of hospitals. On the other hand, preparedness is the only way to deal with these situations, which can subsequently lead to better services and prevent or reduce the number of injuries. Hospitals must provide a standard response plan and practical guidelines for the emergency operations of the hospital’s various wards during disasters as well as internal or external events that can affect hospital employees, patients, visitors, and the community [2, 3]. Hospitals should have a well-planned program in place to prepare for and improve their skills in the case of an emergency. In the United States, the American Society of Surgeons founded the Joint Hospital Accreditation Committee with the support of numerous other institutions, including the Canadian Society of Surgeons and the American Nurses Association, to accredit medical facilities and hospitals. One of the most significant standards of this committee is to provide hospital accreditation as well as to design and implement an emergency preparation program [4, 5]. Internal auditing (within the organization) is one method for determining a hospital’s preparedness. Auditing is a quality improvement process that is systematic, independent, and documented in order to uncover and assess evidence using a set of defined and standard criteria and make necessary improvements [6]. Auditing is a component of professional accountability in clinical governance, which holds providers of various levels of health care responsible and accountable for quality services and continual improvement of those services. Clinical auditing should be prioritized in clinical services [7]. Each auditing has a cycle that comprises the following phases: (1) evaluating the status quo (2) establishing and developing standards (3) using standards to compare outcomes (4) implementing interventions and modifying operations based on results (or providing solutions) (5) re-auditing to verify improved operation [8]. The evidence in assessment is used to assess how auditing criteria are estimated. Auditing should have a clear, independent, systematic, and documented goal, and it should give good recommendations on disaster management approaches, mechanisms, and instruments. Weaknesses, opportunities, threats, and risks can be found during a disaster management audit, which raises managers’ awareness of disaster management, prevents duplication of effort, and ensures the successful implementation of disaster management programs [9]. Recently, total quality management (TQM) has become one of the most effective and extensively used techniques for improving organizational processes on a national and even international scale. FOCUS PDCA, a key TQM tool, is a scientific, logical, and practical method for process improvement that includes a comprehensive and appropriate management toolset for resolving organizational difficulties with the assistance of process owners [10]. Previous studies have mainly assessed the vulnerability of hospitals, but the present study has specifically addressed functional readiness with a standardized tool, and nearly no health-related study documented functional preparedness and audit of hospitals in response to disasters. Furthermore, military hospitals are on the front lines of reacting to disasters, and the selected hospital is one of the largest military hospitals, allowing for a clear picture of its potential and actual capabilities. As a result, the current study aimed to audit the functional preparedness of the chosen military hospital in response to disasters.

## Materials and methods

**Fig. 1:**
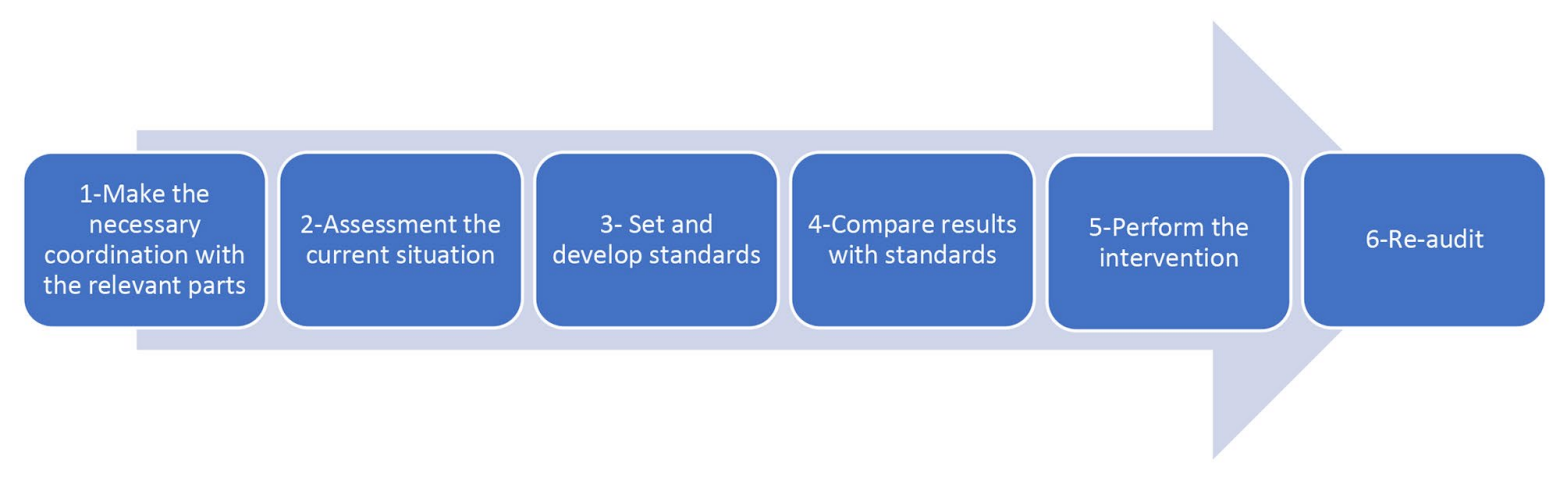
Algorithm of the research steps in studying the audit of functional preparedness of the selected hospital in response to disasters and incidents

Fig. 1 demonstrated the research steps in auditing of functional preparedness of the selected hospital in response to disasters and incidents.

This applied action research was conducted in all wards of selected military hospitals from September 2020 to September 2021. In the fourth auditing phase, functional preparedness training program was considered as the intervention. In this research, a 250-item functional preparedness checklist designed by Heidaranlo (2018) [11]. was used to assess functional preparedness of hospitals during disasters. The checklist’s score ranges from one to five, including poor, very poor, moderate, good, very good evaluation [8]. This checklist has 17 domains: command and control (17 items), 2- risk assessment (9 items), 3- rapid warning system (9 items), 4- capacity building (20 items), 5- constant vital services (18 items), 6- hospital incident command system (28 items), 7- safety/security (14 items), 8- communications (10 items), 9- triage (10 items), 10-hospital evacuation plan (10 items), 11- dead bodies (5 items), 12- logistics support management (36 items), 13- human resource (16 items), 14- monitoring of the epidemiological care system of the hospital (26 items), 15- post-disaster recovery (12 items), 16- cultural considerations ( 8 items), and 17- conclusion and the final question (Table 1). SPSS22 and descriptive statistics were also used for statistical analysis of data.

**Table 1:** level of hospital functional preparedness in the face of disasters based on the score obtained

Level of functional readiness of the hospital	Hospital preparedness percentage	Hospital score obtained
Very weak (1)	0–20%	0-180
Weak (2)	21–40%	181–360
Moderate (3)	41–60%	361–540
Good (4)	61–80%	541–720
Very Good (5)	81–100%	721–900

### The validity and reliability of the instrument

The questionnaire’s face validity, content validity, construct validity, internal consistency and stability were measured in 50 hospitals. The qualitative face validity test was carried out with the help of 15 disaster experts. Ten personnel from the hospitals’ disasters committee assessed the quantitative face validity of the questionnaire. A panel of fifteen specialists assessed the qualitative and quantitative content validity. Known group comparison (KGC) or differential validity has been used for construct validity. Internal consistency using Cronbach’s alpha was applied to assess the questionnaire’s reliability. Cronbach’s reliability coefficient results indicated that the questionnaire was reliable (α = 0.986).

### Functional preparedness-training program

First, a number of health disaster management experts were interviewed and then a functional preparedness-training program and incident command system were designed. Face validity was used to perform the educational content validity. The content was provided to 15 experts in health disaster management and their opinions on the content were obtained by the Delphi method. Results obtained from three rounds of the Delphi showed the validity of standard guidelines for functional preparedness in disasters. In Table 2 The process of reaching topics of standard guidelines for functional preparedness in disasters is shown using the multi-step Delphi technique.

**Table 2:** Summary of topics agreed by the panel of disaster experts regarding functional preparedness

The first round of the Delphi technique	Second round of Delphi technique	Third round of Delphi technique	The final stage of the Delphi technique
Content titles suggested by experts	Content titles suggested by experts	Content titles suggested by experts	
Legal requirements and upstream documents	Legal requirements and upstream documents	HICS (Incident Command System)	HICS
Early Warning System	Command and Control	Command and Control	Command and Control
Strengthening surfaces and walls	Risk Assessment	Risk Assessment	Risk Assessment
Fire risk management	Early Warning System	Early Warning System	Early Warning System
Command and Control	Surge Capacity	Surge Capacity	Surge Capacity
Staff	Continuation of vital services	Continuation of vital services	Continuation of vital services
Communication	Staff	Staff	Staff
Safety of medical gases	safety and security	safety and security	safety and security
Triage	Communication	Communication	Communication
Safe hospital evacuation Plan	Triage	Triage	Triage
Management and support	Safe hospital evacuation Plan	Safe hospital evacuation Plan	Safe hospital evacuation Plan
Methods of transporting the injured in an emergency evacuation	Corpses and the dead	Corpses and the dead	Corpses and the dead
Emergency Medical Teams	Management and support	Management and support	Management and support
Resilience and continuity of vital hospital services	Monitoring of Hospital surveillance System	Monitoring of Hospital surveillance System	Monitoring of Hospital surveillance System
Hospital Surge Capacity	Post-disaster Recovery	Recovery	Recovery
Preparedness in CBRNE Incidence	Cultural Consideration	Cultural Consideration	Cultural Consideration
Field / Mobile Hospital	-	-	-
Management Of Exercises	-	-	-

- In the first stage of the Delphi technique, a supervisor was selected from the research team.
- In the second stage, a panel of experts in disasters was identified.
- In the third stage, standard guidelines for functional preparedness in disasters were raised for the panel of experts. The general questions were presented in the form of a survey, the outlines were described, and the main views of the experts on the standard guidelines for functional preparedness in disasters were extracted, and minor comments were left out. (The first round of the Delphi method)
- In the fourth stage, according to the experts’ answers to the questions of the first round, more explanation was given so that the experts could explain the issues explicitly. The results were collected, unimportant criteria were separated and common responses were extracted. (To remove unimportant criteria in the 5-point Likert scale, the mean score below 3 was removed) (The second round of the Delphi method).
- In the fifth stage, the third round of questions was asked. This round was the decision-making stage about the standard guidelines for functional preparedness in disasters. At this stage, the research team focused more on the joint points and the indicators were screened again. (The third round of the Delphi method)
- In the sixth stage, the final instruction, including the titles and content, were presented to the panel of experts and final agreement was reached.

### The procedure

In the first phase, the military hospital agreed to cooperate in the project. Then, the letter of introduction was received from the university, delivered to the head of the hospital and the necessary coordination with the crisis management department of the hospital was done.

The following steps were made to implement the project in accordance with the audit cycle of hospital functional preparedness:

According to the first phase of the auditing, evaluating the status quo, the functional preparedness of the hospital in response to disasters was determined based on 17 domains of the functional preparedness checklist and the preparedness score of each domain was extracted (The first phase of the auditing).

To evaluate the status quo of the selected hospital, the researcher first collected basic information with the agreement of the senior officials of the hospital and with the help of the managers of the hospital risk committee. The documents of the risk committee pertaining to each of the 17 domains of the hospital’s functional preparedness in response to disasters were reviewed. The final score for the first phase of the auditing was extracted and recorded based on the scores obtained in this phase.

The second and third phases of the auditing are determining standards, and comparing the status quo with the standard one, respectively. The functional preparedness checklist is the only tool that have been made with a scientific and standard instrumentation and a hybrid method with a focus on Text-Context and its psychometrics has been approved, so the score obtained for each item is the standard functional preparedness for that item.

The scores obtained in the second and third phases of the audit cycle (determining the standards, and comparing the status quo with the standard one) were compared with the standard scores of those domains.

In accordance with the fourth phase of the auditing, performing the intervention, the standard guidelines for functional preparedness in disasters were developed for the process owners (managers, healthcare employees and administrative ones) based on the Delphi method; it was trained according to the FOCUS-PDCA model, and appropriate solutions were provided.

According to the fifth phase of the auditing, re-auditing, the functional preparedness score of the selected military hospital in response to disasters was extracted after the intervention, and compared with the standard score before and after preparedness. The information obtained from the hospital evaluation was analyzed using SPSS.

### Intervention

Some domains were moderate, poor and very poor in the standard guidelines for functional preparedness in disasters, so necessary training as well as instruction of FOCUODCA model were done in forms of conferences, feasibility study classes, and the use of visual and auditory resources. Standard guidelines were trained to managers, healthcare staff, and other personnel (procurement officer, security guard, installation workers, so on) in 10 two-hour sessions in the conference hall of the hospital according to the content of the booklet. In addition, the necessary training was provided for the managers and staff of each department, focusing on the weaknesses of that domain (standard deviation).

In order to continue the educational content, an educational booklet designed by the researcher and approved by panel of experts in the field of health and disasters was distributed among managers and officials. The booklet included general information about the incident command system and accreditation items and modifiable points in the mentioned domains. In addition, three groups were formed in WhatsApp according to the levels of the participants to answer their questions and ambiguities. The reports were tracked virtually. The researcher, together with the hospital’s risk committee team visited all wards of the hospital and reminded them of the contents according to their expertise. Then, with the cooperation of the staff of each ward, the FOCUODCA model was implemented to correct the existing weaknesses, focusing on the standard guidelines for the functional preparedness according to the audit cycle (performing the intervention and presenting the solution). In this phase of the audit cycle, the hospital’s risk management committee with the help of the process owners of each part implemented the developed program (51 cases), resource provision (12 cases), training of the developed program (cases 7), tabletop practice (3 cases) and the exercised program revision (1 cases). Table 3 shows the interventions performed on deviant indicators to reach the desired standards.

**Table 3:** Indicators of deviation from the standard and intervention in accordance with the standard of functional preparedness in 17 areas of the tool based on the FOCUS-PDCA model

Domain	Standard deviations	Interventions based on FOCUS-PDCA model and standard functional preparedness
**Command & control**	- making supervisors prepared for commanding incidents in non-working hours. - Lack of active internet and computer equipment in EOC of the hospital - Lack of important information about operations in disasters in EOC	- Resources were supplied by the hospital’s head - The risk management committee conducted training sessions for supervisors. - The risk management committee provided active internet and computer in EOC - EOC was equipped to: Important maps Essential contact list Operational team lists Personnel recalling process
**Risk assessment**	- Recognition of hazards based on their likelihood	- Categorization of likelihood of hazards (high, moderate and poor likelihood) - Prediction of intensity of each hazard and identification of probable injuries
**Early warning system**	- Emergency response plan to hazards - Informing of the occurrence of hazards	- A plan was developed and resources were supplied. - Training was provided. - A plan was developed, which included some correct distribution indices. - People at risk were informed. - Personnel realize alarms and warnings. - Warnings are clear, practical with proper information for a proper response.
**Surge Capacity-**	- Lack of a plan to increase capacity for additional personnel - Lack of a plan for creating master station in each ward for emergencies - Lack of development of service prioritization process - Lack of a plan to provide temporary hospital facilities, meet various needs of survivors in disasters	- A plan was developed and trained. - The plan was assessed in different exercises (operational & tabletop) - A plan was developed to create a master station (at least one room) in each ward for emergencies - It was trained how to direct patients and personnel to these spaces. - Unnecessary services such as some surgical wards and elective surgeries were cancelled. - The process owner implemented a tabletop exercise to prioritize services. - A plan was developed for providing temporary hospital facilities and an on-call program for physicians, nurses, practical nurses and housekeeping aides.
**Continuity of function**	- Lack of a plan to monitor readiness of hospital powerhouse in emergencies - Lack of a plan to monitor fire extinction & detection system in some parts of hospital in emergencies	- Adjusting and updating checklist regarding contact numbers of fuel supply centers, maintenance & repair companies of hospital installations - A plan for producing dual fuel system in hospital - A plan to create fire detection system in CSR, laundry, dining area of the hospital
**Hospital incident command system**	- No substitute for public information office and safety officer in HICS chart	- A substitute for public information officer and safety officer was determined.
**Safety**	- Lack of a plan to safely evacuate and restart the hospital’s laboratory - Lack of a plan to safely evacuate and restart the hospital’s radiology department - Lack of a plan to safely evacuate and restart the hospital’s operating room	- A plan was developed to evacuate safely and restart the hospital’s laboratory. - A plan was developed to safely evacuate and restart the hospital’s radiology department - A plan was developed to evacuate safely and restart the operating room of the hospital
**Security**	- Lack of a plan to inform hospital staff and clients of access restrictions. - Lack of a plan to control crowded areas and parking lots of the hospital. - Lack of a plan to control overcrowding in the hospital.	- A plan was developed to inform the change of route of employees and clients and vehicles at the time of incidents. - Signs were installed inside and outside the hospital indicating that the doors would not open or where new entrances were located. - A program was developed to control crowded areas and parking lots in the hospital. - A program was developed to control the crowds of patients referred to the hospital.
**Communication**	- Lack of a plan to document communications in an emergency - Lack of a plan for communications outside the hospital	- Telephone call recording system application was launched. - Forms were provided to set up minutes. - An individual was designated as a spokesperson to coordinate all hospital communications with the community, media and health authorities. - The spokesperson was trained on security issues due to the military nature of the hospital.
**Triage**	- Not appointing an experienced person to supervise all stages of triage - Determining the entry and exit routes to/from the triage	- An emergency physician was appointed to supervise the triage process. - A general surgeon was appointed as the first substitute in the triage. - In the absence of an emergency physician or general surgeon in triage, the emergency clerk will be responsible for supervising the triage process. - Specific entry and exit routes and necessary training were given to the process owners (nurses, ambulance drivers, assistant nurses, security guards, porters, etc.)
**Safe evacuation of the hospital**	- Lack of a plan regarding signs in emergencies - Lack of a plan for elevators in an emergency - Lack of a plan for horizontal evacuation in an emergency - Lack of a plan for vertical evacuation in an emergency	- Maps of floors were installed in each floor. - Maps clearly show the emergency exits of the building. - The route of the emergency staircase is clearly shown. - Familiar and clear terms are used in the maps to find the emergency exits. - Instructions for using the elevator in case of fire or emergency evacuation were inserted inside and on the elevators - Signs were installed for the emergency exit stairway. - The horizontal evacuation plan of the hospital was developed and trained, meaning that everyone in each department had to move to the opposite side of the danger. - The vertical evacuation plan of the hospital was developed: - This refers to the complete evacuation of a floor. - If the exact location of incident was specified, staff would be relocated to another location in the building where safety has been determined (at least two floors below the incident site). In the event of a complete evacuation of a structure, staff must be relocated to a safe area outside the building.
**Dead bodies**	- Lack of a plan to make a temporary mortuary in an emergency - Lack of a plan on how to discharge the deceased from the mortuary in an emergency	- A plan was developed to make a temporary mortuary with 4 parts (reception room, corpse viewing room, a place for keeping corpses that are not suitable for examination and a room for archiving files and personal belongings). - Discharge of the deceased from the mortuary in an emergency includes: - The documents requested for the discharge of dead bodies. - The person to whom the deceased should be delivered legally. - Legal confirmation of death, preparation of official death report and return of belongings to people close to the deceased.
**Financial/support management/ agreements**	- Lack of a plan to identify disasters team members based on tasks assigned in an emergency - Lack of a plan to provide public liability insurance in case of emergency • Lack of a plan to conclude an agreement with public hospitals in case of emergency Lack of a plan to conclude an agreement with military hospitals in an emergency - Lack of a plan to conclude an agreement with equipment and pharmaceutical companies in case of emergency - Lack of a plan for tabletop exercise under the supervision of the incident and disaster committee in the hospital, according to the program notified by the Ministry of Health	- Covers were prepared based on the division of the various positions of the disaster committee chart (commander, senior officers, operations, planning, finance and support). - The color of the covers indicates the type of task assigned. - A plan was developed to access the covers in the first hour of the incident. - An initial plan was developed in order to provide public liability insurance in case of emergency. - An initial plan was developed to conclude an agreement with public hospitals in an emergency - An initial plan was developed to conclude an agreement with military hospitals in an emergency - An initial plan was developed to conclude an agreement with equipment and pharmaceutical companies in case of emergency. Roundtable training program was developed for senior managers.
**Human resource**	- Lack of a plan to train personnel to play their role in the face of disasters - Lack of a plan to take care of staff’s families (children, patients, older adults and disabled members) in an emergency - Lack of a plan for employment of support staff and volunteers at the time of the incident. - Lack of a plan to complete the personnel injury form and follow up reports in an emergency	- The plan of prioritization and needs assessment of educational priorities was developed. - An initial plan was developed to hold related training classes in the next 6 months. - As staff were working longer hours, an initial plan was developed to take care of the staff’s families (children, patients, older adults and disabled members). - Necessary communications were established to allocate the required spaces to this plan. - An initial plan was developed to describe the duties and how to use the volunteers. - An initial plan was developed to ensure the basic needs of volunteers in an emergency (resting place, food, etc.) for at least 72 h. - The process of temporary liability insurance was developed for volunteers. - The process of completing the personnel injury form and following up reports was developed.
**Epidemiology**	• Lack of a plan to replace the hospital’s food cold storage in case of emergency. -Failure to implement a plan to control the drinking water of the hospital in an emergency - Lack of a plan to replace the hospital wastewater treatment system in case of damage - Lack of a plan for regular chlorination of drinking water and accurate recording of chlorine in the water in disasters - Lack of a plan to replace the hospital waste incinerator system in the event of damage in an emergency. - Lack of a plan for continuation of hospital laundry in case of damage in an emergency. - Lack of a plan for continuation of hospital CSR in case of damage in an emergency	• In case of damage to the hospital food cold storage, an alternative plan was developed. - A plan was developed, trained, exercised and revised to control the drinking water of the hospital and the water storage tanks were supplied in case of damage. - A plan was developed to replace the hospital wastewater treatment system. - A plan was developed for regular chlorination of drinking water and recording of the exact amount of chlorine in the water. - A plan was developed to replace the hospital incinerator system. - A plan was developed to continue the hospital laundry in case of damage in an emergency, - A plan was developed to continue the hospital CSR in case of damage in emergency situations,
**Post-disaster recovery**	- Lack of psychological support teams to respond to incidents and disasters - Lack of mental health support program for staff and their families in the short and long term in emergencies. - Lack of a mental health support program for the injured and their families in the short and long term in emergencies. - Lack of a plan for job description of volunteers in the recovery phase in case of emergency.	- An initial plan was developed to form psychological support teams to respond to incidents and disasters. - According to the standard, teams consisting of social workers, psychological counselors and clergymen were defined in the initial plan. - An initial plan was developed to support mental health of staff and their families in short-term courses with the presence of mental health counselors. - An initial plan was developed to support the mental health of the injured and their families in short-term courses with the presence of mental health counselors. - An initial plan was developed for job description of volunteers in the recovery phase.
**Cultural considerations**	- Lack of evaluation plan regarding provision of healthcare services based on religious norms in case of emergency - Lack of a plan to create a space for the religious duties of the injured, their companions and staff (taking into account the religious norms) in case of emergency. - Lack of plan for welcoming VIPs in case of emergency - Lack of a plan to engage clergymen in disasters phases in emergencies.	- An evaluation checklist was prepared to provide healthcare services based on religious norms in case of emergency. - An initial plan was developed to create a space for the religious duties of the injured, their companions and staff (taking into account the religious norms) in an emergency. - An initial plan was developed for welcoming VIPs in case of emergency. Description: According to the readiness standard, VIP refers to high-ranking government officials, influential and well-known people in the community, so senior hospital managers are obliged to accompany them when visiting the injured and the losses imposed on the hospital. Evaluators were advised to be careful in this regard because Iranian officials have a great desire to be shown on the media, and therefore disaster commanders and efficient forces spend much time reporting to VIPs. Therefore, it is important to have a previous plan to coordinate with VIPs. - In collaboration with the hospital’s doctrinal unit, an initial plan was developed to engage clergymen in disasters Description: * Religious scholars and clergymen are skillful in spiritual matters and can motivate personnel to be prepared in the preparation phase. They spiritually accelerate the return of injured to their daily activities in the recovery phase.

The researcher with the help of the hospital’s risk committee managers re-evaluated the hospital’s functional preparedness one month after training. Finally, the results before and after the intervention were compared and reported according to the fifth phase of the auditing.

## Results

The purpose of analysis in any scientific research is to obtain results and provide scientific suggestions. Therefore, in this study, the results were examined according to the information obtained. In order to describe the data, descriptive statistics was used, and preparedness percent table was used to show study population response rate to each item. The mean score of hospital’s preparedness in response to disasters was 508 out of 900 and the percentage of the hospital’s functional preparedness was 56.44% before the intervention. The mean score of hospital’s preparedness in response to disasters was 561, the percentage of the hospital’s functional preparedness was 63.63% after the intervention, and the information was summarized in the following 17 columns. Then the results were compared before and after the intervention and the functional preparedness of the selected hospital was reported. In Table 4 based on topics and in Table 5 based on total score, hospital functional preparedness in disasters before and after the intervention is reported.

**Table 4:** Assessment of hospital functional preparedness in disasters before and after the intervention

Questionnaire Domains	Maximum tool score	Before intervention Score	After intervention Score	Percentage of preparation before the intervention	Percentage of preparation after the intervention	Functional preparedness level after the intervention
**Command& control**	44	24	27	54%	61%	Good
**Risk assessment**	8	6	7	75%	88%	Very Good
**Early warning system**	18	9	11	50%	61%	Good
**Surge Capacity-**	76	34	43	44%	56%	Moderate
**Continuity of function**	80	54	58	67.5%	72%	Good
**Hospital incident command system**	119	107	110	89%	92%	Very Good
**Safety**	37	21	24	56%	64%	Good
**Security**	44	28	34	63%	77%	Good
**Communication**	40	25	29	62%	72%	Good
**Triage**	30	21	24	70%	80%	Good
**Safe evacuation of the hospital**	36	12	19	36%	52%	Moderate
**Dead bodies**	16	4	8	28%	44%	Moderate
**Financial/support**	135	67	79	49%	58%	Moderate
**Human resource**	46	18	28	38%	66%	Good
**Epidemiological monitoring**	98	45	57	45%	58%	Moderate
Recovery	44	18	24	41%	54%	Moderate
**Cultural considerations**	28	13	18	45%	64%	Good

**Table 5:** General (total score) comparison of functional preparedness of the hospital before and after the intervention

	before the intervention	after the intervention
Hospital score obtained	508	561
Hospital readiness percentage	56.44%	62.33%
Level of functional readiness of the hospital	Level three (moderate)	Level four (Good)

## Discussion

​​**Command and control**.

The first domain, command and control, is one of the important elements in hospital’s preparedness for effective response to disasters. The score obtained before the intervention was 54%, which reached 61% after the intervention. Hospital preparedness improved from a moderate to a good level after the intervention. The results of this study are consistent with Karimian’s study (2013), which examined the effect of training hospital preparedness principles according to the national program on the level of disaster preparedness of Shahid Motahari hospital in Tehran. The overall hospital preparedness score changed from 178 to 210 and the hospital had the highest preparedness in terms of command and control and the least preparedness in terms of post-disaster recovery [12]. This result was also consistent with the study of Parsaei et al. (2017) that examined hospital’s disaster preparedness in Mazandaran [13]. Zaboli et al. (2014) showed a low level of command and control in the selected hospitals in Tehran [14], which is inconsistent with the findings of current study. Ingrasia (2016) studied hospital preparedness in the face of incidents in Italy. He reported a high level of command and control, which is consistent with the current study [12].

### Risk assessment

The hospital preparedness was 82% to identify strengths and weaknesses, which reached 90% after the intervention (very good). Amiri et al. (2011) showed that Semnan hospitals were 43.8% ready to reduce structural risks and reported a moderate quality of preparedness in this domain [13]. Zaboli et al. (2014) showed a low level of risk assessment in the selected hospitals in Tehran. The studies done by Amiri et al. and Zaboli et al. are inconsistent with the current study [14].

Other study showed that to increase the current level of disaster preparedness, the main weaknesses of hospitals should be identified and a plan should be developed to remove obstacles and improve structures [15].

### Early warning system of the hospital

The early warning system preparedness of the hospital has been increased from 50% before the intervention to 61% after the intervention, which indicates the hospital’s good preparedness to implement the rapid warning system in disasters. Delshad et al. (2015) conducted a study at Shahid Motahari hospital in Tehran. They indicated a moderate level of disaster preparedness in Shahid Motahari hospital as well as a significant increase in the hospital preparedness level with the implementation of the early warning system [16].

Fallahi et al. (2011) conducted a descriptive field study in a military hospital in Tehran. The overall result regarding ​​the early warning system is lack of preparedness in the face of earthquake [17]. The studies conducted by Delshad et al. and Fallahi are inconsistent with the current study. Assessing the vulnerability of the hospital is one of the strengths of the hospital examined in the current study.

### Capacity building

In terms of increasing the hospital capacity, functional preparedness increased from 44% before the intervention to 56% after the intervention. Despite measures taken and an increase in the score, there was no improvement in functional preparedness level in this domain. This seems to be due to the lack of identification of the potential capacity of the hospital and the non-implementation of the developed plans. This domain was consistent with the study of Parsai et al. on the disaster preparedness of Mazandaran hospitals [13]. Vali et al. (2014) also showed that hospitals affiliated to Tabriz University of Medical Sciences were 47% ready (moderate level) in unexpected events in terms of physical space [18] Also, Hasanpoor et al. (2015), assessed the level of preparedness of Karaj hospitals in terms of surge capacity the average level [19]. In practice, the results of these studies are in line with the results of current research in terms of hospital surge capacity. Factors such as estimating the consumption of resources and essential items, increasing coordination to ensure the provision of resources and reserves and necessities, and concluding an agreement are effective.

### Continuation of vital functions

The hospital score increased from 67 to 72% regarding the constant vital services. The hospital score was good in this domain. The military hospital has proper planning to store the necessary equipment for disasters, which is consistent with the study of Rajabi (2017) on the effects of the rehabilitation hospital’s risk management program during disasters, in which there was an increase in the constant vital services (61%) [20].

### Incident command system

In this domain the hospital score was good and increased from 89 to 92% which is due to the good training of hospital commanders and their management in critical situations due to the military nature of hospital. A good command system can provide disaster preparedness and constructive management in disaster control strategies [21]. Habibzadeh et al. (2015) pointed to the lack of an incident command system in disasters in Seyed Al-Shohada hospital in Urmia [22]0). Their study was not consistent with the current one.

### Safety

The hospital score was good and increased from 56 to 64%. Some processes in the intervention were rewritten and practiced, which increased the score. Some flaws can be modified by correcting the process while others need costs and structural reforms, which should be among the priorities of funding and reforming hospital facilities. In the study of Tabatabai et al. (2016), the readiness of selected hospitals in the field of safety was assessed at a moderate level (52%) [23]. This result was inconsistent with current study, this difference may be due to differences in the type of hospitals selected in the study. Although, the results of Hatami’s study are in line with the findings of our study [24].

### Security and communication

The selected hospital was at a good level in terms of security and communication due to its military system, access to radio and wireless facilities, as well as the multi-layered communication system and a good monitoring structure. This result was inconsistent with the results of Maleki.et.al that examined security level of the hospitals affiliated to Iran University of Medical Sciences using a 6-item questionnaire and they reported a low level of preparedness [25]. However, this result is consistent with the study conducted by Daneshmandi et al. that assessed security preparedness in one of the hospitals in Tehran [26]. Sokhanvar et al. (2015) showed a moderate level of security in selected hospitals in Tehran, which is inconsistent with the current study [27]. The results of a study conducted by Daneshmandi et al. (2014) showed a moderate level of communication in the selected hospital in Tehran (50%) [26]. Zaboli et al. (2014) showed a poor level of communication in selected hospitals in Tehran [14].

### Triage

The hospital score in this domain changed from 70 to 80%. The hospital preparedness score was at a good level due to the construction of a new emergency building and consideration of a physical space of triage and separation of outpatients and admitted ones. The result is consistent with the Parsaei’s study (2019) [13].

According to Janati et al. (2018), the mean emergency response of Karaj hospitals in the face of disasters was 17.44%, which is the highest response among the studied areas related to hospital triage (70.33) [21].

Ezzati et al. (2016) reported poor triage (30.3%) in social service hospitals in Kermanshah, which is inconsistent with the current study [28].

### Dead bodies

The hospital score was poor in this domain (28%), which was average after the intervention (44%). The reason is that there is no person in charge of mortuary, and the hospital is not ready to manage dead bodies. A review of the literature and studies on hospital preparedness in disasters revealed that previous studies did not mention the domain of ​​dead bodies and the hospitals were not measured in this regard.

### Human resource

The hospital obtained a good score in this domain (from 38 to 66%). Salari et al. (2011) in Shiraz ,Hojjat (2009) in Tehran and Vali et al. (2014) in Tabriz reported a good level of preparedness in terms of human resource, which is consistent with the current study [18, 29, 30] [29]. Zaboli et al. (2014) showed poor human resource in selected hospitals in Tehran, which is not consistent with the current study [14].

### Financial and resource support management

The hospital score was 49% before the intervention and reached 58% after the intervention. The score obtained in this domain was moderate. The stronger the hospital support, the lower the casualties due to disasters will be. Necessary plans and programs in this domain were done in the hospital, but they were not operational in the exercise, which is the reason for the hospital’s moderate level. Vali et al. (2014) showed a moderate level of disaster preparedness of the hospitals affiliated to Tabriz University of Medical Sciences in terms of support (57%) [18].

Daneshmandi et al. (2014) reported a good preparedness level of the selected hospital in Tehran in terms of support (64.3%) [26]. According to various studies, in order that a hospital is fully prepared to respond effectively to an incident, specific policies in the development of clear guidelines and protocols play an important role in achieving this preparedness.

### Post-disaster recovery

The hospital scores in this domain were 40% and 54% before and after the intervention respectively, which were at a moderate level. According to Hassanpour et al. (2015), the mean emergency response of Karaj hospitals in the face of incidents and disasters was 17.44% and the worst aspect of preparedness was related to post-disaster recovery [19]. Karimiyan et al. (2016) reported the lowest level of preparedness in terms of post-disaster recovery in Shahid Motahari hospital in Tehran [12]. It seems that the non-appointment of individuals and also the lack of performing exercise to measure the hospital needs and resources, as well as reconstruction in cases of evacuation is one of the improvable items in this area [20].

### Epidemiology

The hospital preparedness percentage in this domain was 45% before the intervention, which reached 58% after the intervention. Amiri et al. (2011) reported a moderate level of hospital preparedness in terms of epidemiology (56.2%) [31]. Mortelmans et al. (2014) reported poor preparedness of selected Belgian hospitals in terms of epidemiology [32]. The study conducted by Amiri et al. (2011) is consistent with the current study and the study of Mortelmans is inconsistent with the current study.

### Cultural considerations

The hospital preparedness percentage in this domain was 46%, which increased to 64% after the intervention (a good level). A review of the literature and studies on disaster preparedness in hospitals revealed that previous studies did not address cultural considerations and that hospitals were not measured in this regard.

## Conclusions and suggestions

The results of the present study show that staff training along with auditing of the studied areas can increase the staff preparedness and identify the strengths and weaknesses of hospitals. Hospitals must be ready in all areas to respond to hazards effectively. To improve the level of preparedness, managers must pay more attention to areas that received lower scores. In order to ensure the effective response of hospitals to all possible hazards, they must be ready on a continuous basis, so it is recommended to assess the hospital preparedness annually.

## Data Availability

The datasets used and analyzed during the current study available from the corresponding author on reasonable request.
